# Excessive Osteocytic Fgf23 Secretion Contributes to Pyrophosphate Accumulation and Mineralization Defect in Hyp Mice

**DOI:** 10.1371/journal.pbio.1002427

**Published:** 2016-04-01

**Authors:** Sathish K. Murali, Olena Andrukhova, Erica L. Clinkenbeard, Kenneth E. White, Reinhold G. Erben

**Affiliations:** 1 Department of Biomedical Sciences, University of Veterinary Medicine, Vienna, Austria; 2 Department of Medical and Molecular Genetics, Indiana University School of Medicine, Indianapolis, Indiana, United States of America; Royal Veterinary College London, UNITED KINGDOM

## Abstract

X-linked hypophosphatemia (XLH) is the most frequent form of inherited rickets in humans caused by mutations in the phosphate-regulating gene with homologies to endopeptidases on the X-chromosome (*PHEX*). *Hyp* mice, a murine homologue of XLH, are characterized by hypophosphatemia, inappropriately low serum vitamin D levels, increased serum fibroblast growth factor-23 (Fgf23), and osteomalacia. Although Fgf23 is known to be responsible for hypophosphatemia and reduced vitamin D hormone levels in *Hyp* mice, its putative role as an auto-/paracrine osteomalacia-causing factor has not been explored. We recently reported that Fgf23 is a suppressor of tissue nonspecific alkaline phosphatase (*Tnap)* transcription via FGF receptor-3 (FGFR3) signaling, leading to inhibition of mineralization through accumulation of the TNAP substrate pyrophosphate. Here, we report that the pyrophosphate concentration is increased in *Hyp* bones, and that *Tnap* expression is decreased in *Hyp*-derived osteocyte-like cells but not in *Hyp*-derived osteoblasts ex vivo and in vitro. In situ mRNA expression profiling in bone cryosections revealed a ~70-fold up-regulation of *Fgfr3* mRNA in osteocytes versus osteoblasts of *Hyp* mice. In addition, we show that blocking of increased Fgf23-FGFR3 signaling with anti-Fgf23 antibodies or an FGFR3 inhibitor partially restored the suppression of *Tnap* expression, phosphate production, and mineralization, and decreased pyrophosphate concentration in *Hyp*-derived osteocyte-like cells in vitro. In vivo, bone-specific deletion of *Fgf23* in *Hyp* mice rescued the suppressed TNAP activity in osteocytes of *Hyp* mice. Moreover, treatment of wild-type osteoblasts or mice with recombinant FGF23 suppressed *Tnap* mRNA expression and increased pyrophosphate concentrations in the culture medium and in bone, respectively. In conclusion, we found that the cell autonomous increase in Fgf23 secretion in *Hyp* osteocytes drives the accumulation of pyrophosphate through auto-/paracrine suppression of TNAP. Hence, we have identified a novel mechanism contributing to the mineralization defect in *Hyp* mice.

## Abstract 2

A novel mechanism involving autocrine and paracrine actions of fibroblast growth factor-23 contributes to the mineralization defect observed in *Hyp*, a mouse model for X-linked hypophosphatemia.

## Author Summary

X-linked hypophosphatemia (XLH) is the most frequent form of inherited rickets in humans. A mouse model of XLH, known as *Hyp*, is characterized by exceptionally low serum phosphate and vitamin D levels, increased serum levels of the hormone fibroblast growth factor-23 (Fgf23), and impaired bone mineralization. Fgf23 is secreted from two classes of bone cells known as osteoblasts and osteocytes. Fgf23 increases urinary phosphate excretion and suppresses vitamin D hormone production in the kidney. Although Fgf23 is known to be responsible for lower blood phosphate and vitamin D hormone levels in *Hyp* mice, its putative role as a signaling factor causing impaired mineralization has not been explored. We recently reported that Fgf23 is a suppressor of tissue nonspecific alkaline phosphatase (*Tnap)* gene expression via FGF receptor-3 (FGFR3) signaling in osteoblasts, leading to inhibition of mineralization through accumulation of the TNAP substrate pyrophosphate. Pyrophosphate is a potent inhibitor of mineralization. Using a combination of cell culture and animal models, we report that the increase in osteocyte Fgf23 secretion of *Hyp* mice leads to FGFR3-mediated suppression of TNAP with subsequent accumulation of pyrophosphate. Hence, we have identified a novel signaling mechanism by which excessive osteocytic secretion of Fgf23 contributes to the mineralization defect in *Hyp* mice.

## Introduction

X-linked hypophosphatemia (XLH) is the most frequent form of inherited rickets in humans. XLH is caused by inactivating mutations in the phosphate-regulating gene with homologies to endopeptidases on the X-chromosome (*PHEX*) [1–3]. Similarly, a loss-of-function deletion in *Phex*, the murine homologue of *PHEX*, leads to an XLH-like phenotype in *Hyp* mice, a well-known animal model for XLH [4–6]. *PHEX*/*Phex* is predominantly expressed in bone and teeth and at lower levels in muscle, skin, brain, and lungs [7,8]. Both XLH patients and *Hyp* mice are characterized by hypophosphatemia, impaired bone mineralization, inappropriately low serum vitamin D hormone (1,25(OH)_2_D_3_), and increased circulating intact fibroblast growth factor-23 (FGF23) [9–11]. FGF23 is a phosphaturic hormone, mainly produced by osteoblasts and osteocytes in response to increased extracellular phosphate and circulating 1,25(OH)_2_D_3_ [12]. In renal proximal tubules, FGF23 suppresses the membrane expression of the type II sodium-phosphate cotransporters Npt2a and Npt2c, which are necessary for the urinary reabsorption of phosphate [13]. In addition, FGF23 suppresses the renal proximal tubular expression of 1α-hydroxylase [14], the key enzyme responsible for vitamin D hormone production. Fgf23 requires the obligatory coreceptor *α-Klotho* (Klotho) to bind to the ubiquitously expressed fibroblast growth factor receptor 1c (FGFR1c) [15,16]. Hence, the hormonal actions of Fgf23 are restricted, at least at physiological concentrations, to tissues expressing Klotho such as proximal and distal tubules in the kidney, parathyroid gland, choroid plexus in the brain, and sinoatrial node in the heart [13,17].

The molecular mechanisms why loss of *PHEX*/*Phex* function leads to increased FGF23 secretion in osteoblasts and osteocytes are still incompletely understood. PHEX is an ectoenzyme thought to be involved in the proteolytic processing of extracellular matrix (ECM) proteins. Earlier studies in *Hyp* mice revealed aberrant processing of SIBLING (Small Integrin-Binding Ligand, N-linked Glycoprotein) proteins such as matrix extracellular phosphoglycoprotein (MEPE) [18], causing accumulation of acidic serine- and aspartate-rich MEPE-associated motif (ASARM) peptides. ASARM peptides are potent inhibitors of mineralization, and are thought to be at least partially responsible for the mineralization defect observed in *Hyp* mice [19]. Another substrate of PHEX is the ECM protein osteopontin (OPN), a well-known mineralization inhibitor that binds to hydroxyapatite (HA) crystals and blocks the deposition of HA onto ECM [20]. In addition to ASARM peptides, OPN was shown to be increased in bones of *Hyp* mice [21]. Because the mineralization defect present in Dentin matrix protein-1 (*Dmp-1*)-deficient mice also leads to overexpression of Fgf23 [22], it is currently thought that osteocytes respond to impaired mineralization by increased Fgf23 secretion. It is interesting to note in this context that ablation of Fgfr1 in bone partially rescues the excessive Fgf23 secretion in *Hyp* mice, suggesting that Fgfr1-mediated signaling may somehow be involved in the mechanism how osteocytes sense mineralization in the surrounding matrix [23]. In addition, long-term inhibition of FGFR by a pan-FGFR inhibitor in *Hyp* and *Dmp-1-*deficient mice leads to normalization of serum phosphate and calcium and improves mineralization [24]. Besides disturbed mineralization, defective phosphate sensing in osteoblasts has also been implicated to play a role in the augmented Fgf23 secretion in *Hyp* mice [25].

Although the exact mechanism driving Fgf23 secretion in *Phex* and *Dmp-1*-deficient models has remained elusive thus far, several lines of evidence suggest that increased circulating Fgf23 is a major pathogenetic factor in XLH patients and *Hyp* mice, leading to hypophosphatemia and subsequently impaired bone mineralization. Firstly, extraskeletal overexpression of FGF23 also causes hypophosphatemia and osteomalacia [26,27]. Secondly, ablation of *Fgf23* in *Hyp* mice recapitulates the *Fgf23*-null phenotype [28,29]. Thirdly, treatment of *Hyp* mice with anti-Fgf23 antibodies normalizes serum phosphate and vitamin D hormone levels, decreases osteoid volume, and improves bone mineralization [9,15,24]. All these findings suggest that excessive Fgf23 secretion is the major driving force behind the *Hyp* phenotype.

However, because osteoblasts isolated from *Hyp* mice fail to mineralize in a normal fashion in vitro [30], and dietary phosphate supplementation attempting to correct hypophosphatemia did not rescue the osteomalacia in *Hyp* mice [31], it is likely that the mineralization defect in *Hyp* mice has at least two components, namely systemic hypophosphatemia plus independent alterations in the ECM [32]. The relative contribution of local accumulation of ASARM peptides and of OPN in the ECM versus the endocrine phosphaturic effect of Fgf23 to the osteomalacia observed in *Hyp* mice is currently unclear.

We recently discovered that FGF23 suppresses tissue nonspecific alkaline phosphatase (TNAP) transcription and leads to decreased local inorganic phosphate (Pi) production as well as accumulation of pyrophosphate (PPi) by a Klotho-independent, FGFR3-mediated signaling axis in osteoblasts [33]. PPi is another well-known inhibitor of mineralization produced by osteoblasts and osteocytes. Increased levels of PPi in the ECM are known to impair the mineralization process by binding to HA crystals [34–36]. Conversely, absence of PPi in the ECM either via genetic ablation of its intracellular-to-extracellular transporter progressive ankylosis (ANK) [37] or ablation of ectonucleotide pyrophosphatase/phosphodiesterase 1 (ENPP1) [38], an enzyme which produces PPi from ATP, results in hypermineralization of bones. Increased levels of PPi in the ECM can be a consequence of two different mechanisms, either increased production and transportation of PPi to the ECM, or decreased hydrolysis of PPi by TNAP, leading to accumulation of PPi in the ECM.

Based on our recent finding that Fgf23 is a regulator of *Tnap* transcription, we hypothesized that excessive Fgf23 secretion in *Hyp* osteocytes could locally contribute to defective mineralization by suppressing TNAP and increasing PPi concentrations. Here, we report that the PPi concentration is indeed increased in *Hyp* bones, and that *Tnap* expression is decreased in *Hyp*-derived osteocyte-like cells ex vivo and in vitro. In addition, we show that blocking of increased Fgf23-FGFR3 signaling in *Hyp*-derived osteocyte-like cells partially restores the suppression of TNAP expression, phosphate production, and mineralization in vitro. Thus, we have identified a novel mechanism contributing to the defective mineralization in *Hyp* mice.

## Results

### PPi Concentration Is Increased in Bones of *Hyp* Mice

**Fig 1:**
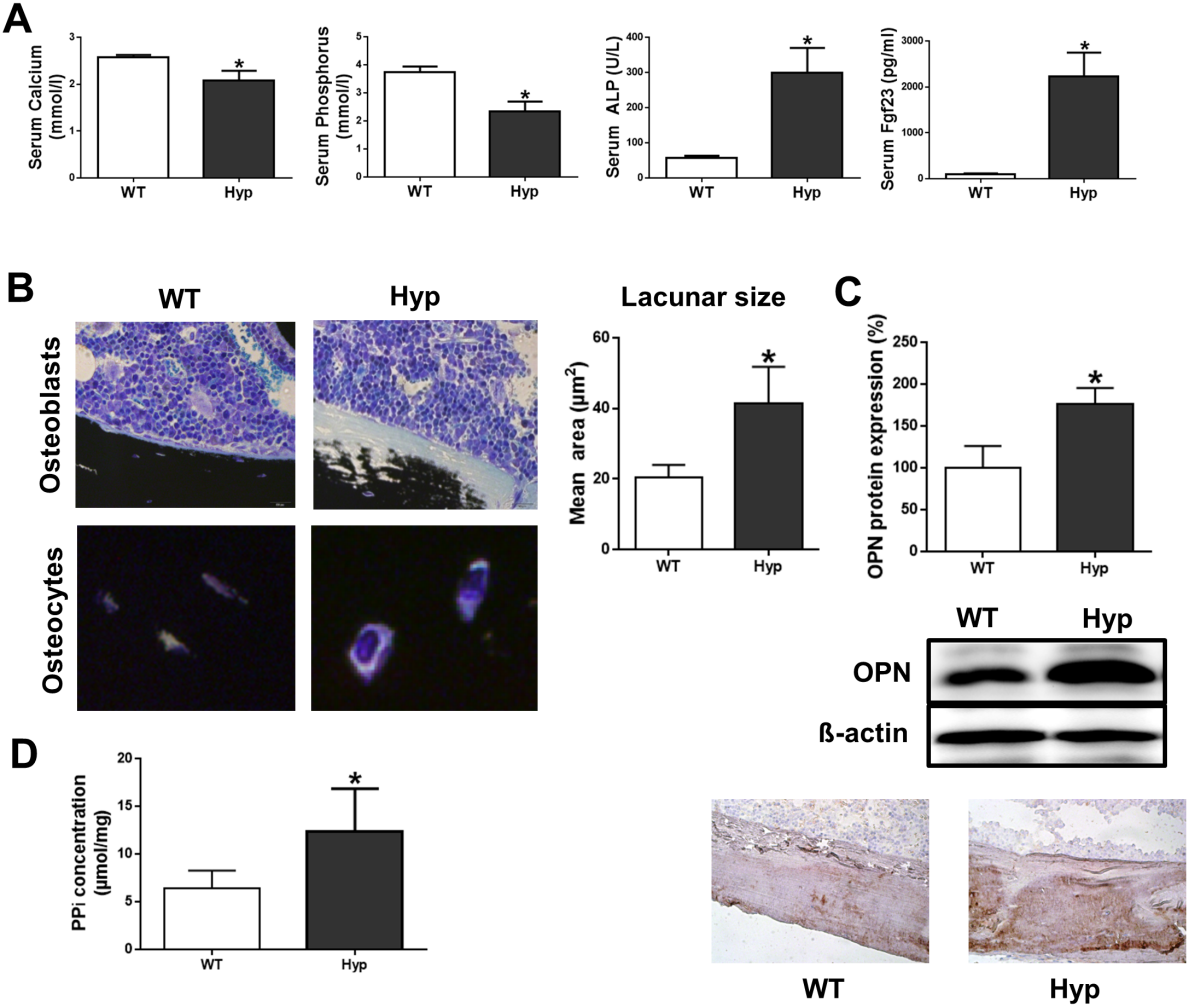
PPi concentration is increased in femurs of *Hyp* mice. **(A)** Serum calcium, phosphate, ALP activity, and intact Fgf23 in 3-mo-old male WT and *Hyp* mice. **(B)** Von Kossa/McNeal staining of 3-μm-thick undecalcified plastic sections of distal femurs from 3-mo-old male WT and *Hyp* mice and quantification of mean area of osteocytic lacunae. **(C)** Quantification of OPN protein expression by western blotting of proteins isolated from femurs (upper panels) and immunohistochemical staining of OPN protein expression in femoral cortical bone (lower panels) in 3-mo-old male WT and *Hyp* mice. **(D)** PPi concentration in extracts of whole femurs from 3-mo-old male WT and *Hyp* mice. Each data point is the mean ± standard deviation (SD) of four mice. Individual values are given in S1 Data. *, *p* < 0.05 versus WT.

It is well known that *Hyp* mice are characterized by hypophosphatemia, hypocalcemia, impaired bone mineralization, and increased serum Fgf23 [9]. This was confirmed in our study. Three-month-old male *Hyp* mice used in our experiments were hypophosphatemic and hypocalcemic, exhibited elevated serum alkaline phosphatase (ALP) activity and increased serum intact Fgf23 (Fig 1A) and showed impaired bone mineralization as evidenced by widened osteoid seams and enlarged osteocyte lacunae in histological bone sections (Fig 1B). Since it was previously reported that OPN protein expression is increased in *Hyp* mice [21], we quantified OPN protein expression in femur extracts from wild-type (WT) and *Hyp* mice by western blotting. As shown in Fig 1C, OPN protein expression was higher in *Hyp* femur extracts compared to WT mice. Immunohistochemistry confirmed increased OPN protein expression in *Hyp* compared to WT bones (Fig 1C, lower panel). According to our hypothesis, the concentration of PPi should be increased in *Hyp* bones. To initially test whether this hypothesis may be worth pursuing, we quantified the amount of PPi in WT and *Hyp* mice femur extracts. As shown in Fig 1D, the PPi concentration in *Hyp* bones was indeed higher compared to WT bones.

### Osteocyte-Rich Cell Fractions Isolated from *Hyp* Femurs Show Increased mRNA Expression of PPi Regulating Genes and Decreased *Tnap* mRNA Expression Ex Vivo

**Fig 2:**
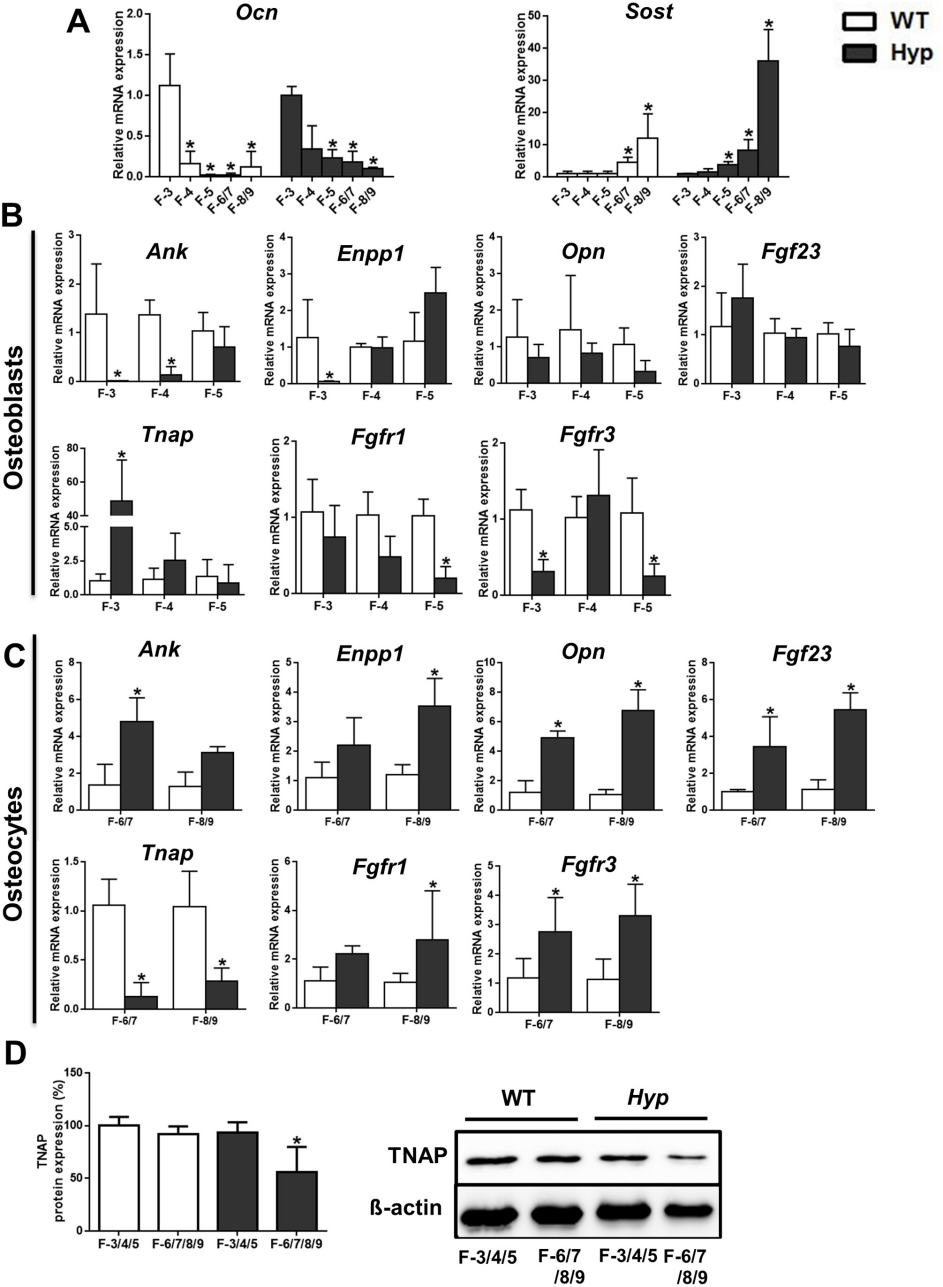
Osteocyte-rich cell fractions isolated from *Hyp* femurs show increased mRNA expression of PPi-regulating genes and decreased mRNA expression of *Tnap* ex vivo. **(A)** mRNA abundance of the osteoblast-specific gene *Ocn* and of the osteocyte-specific gene *Sost* in total RNA isolated from cell fractions harvested by sequential digestion from femurs of 3-mo-old male WT and *Hyp* mice. **(B–C)** *Ank*, *Enpp1*, *Opn*, *Fgf23*, *Tnap*, *Fgfr1*, and *Fgfr3* mRNA abundance in total RNA isolated from osteoblast-rich fractions F-3 to F-5 **(B)** and from osteocyte-rich fractions F-6/7 to F-8/9 **(C)** harvested by sequential digestion from femurs of 3-mo-old male WT and *Hyp* mice. **(D)** TNAP protein expression in pooled osteoblast- and osteocyte-rich fractions harvested by sequential digestion from femurs of 3-mo-old male WT and *Hyp* mice. Each data point is the mean ± SD of four samples from four different mice. Individual values are given in S1 Data. *, *p* < 0.05 versus F-3 in A; *, *p* < 0.05 versus WT in B–C; *, *p* < 0.05 versus WT F-3/4/5 in D.

Our hypothesis predicts that increased Fgf23 secretion in bone cells from *Hyp* mice would suppress TNAP and would subsequently lead to accumulation of PPi. However, in contrast to our hypothesis, serum ALP in *Hyp* was actually higher compared to WT mice (Fig 1A). However, we reasoned that the inhibitory effect of Fgf23 on TNAP might be cell-specific in bone and might only occur in osteocytes where the Fgf23 concentrations in the extracellular fluid are probably highest. To investigate a potential cell-specific effect of Fgf23 on osteoblasts and osteocytes, we isolated osteoblast- and osteocyte-rich fractions from femurs of WT and *Hyp* mice, using a sequential digestion technique [39]. To confirm the successful isolation of osteoblasts and osteocytes, we analyzed the mRNA abundance of the osteoblast-specific marker osteocalcin (*Ocn*) [40], and of the osteocyte-specific marker sclerostin (*Sost*) [41]. Fractions (F) 1 and 2 were discarded because of the high contamination with other cell types. In both WT and *Hyp* mice, F-3 showed higher levels of *Ocn* mRNA expression compared to the other fractions (Fig 2A), suggesting that this was an osteoblast-rich fraction. Conversely, *Sost* mRNA expression was low in F-3, F-4, and F-5, and increased 5- to 40-fold in F-6/7 and F-8/9, respectively (Fig 2A). Based upon these results we considered fractions 3–5 as osteoblast-rich, and F-6/7 and F-8/9 as osteocyte-rich in both WT and *Hyp* femurs (Fig 2A).

Analysis of gene expression in osteoblast-rich fractions revealed lower mRNA expression of *Ank* and *Enpp1* and ~50-fold higher *Tnap* expression in *Hyp*- versus WT-derived F-3 (Fig 2B). The most pronounced differences between WT and *Hyp* mice were observed in F-3. Interestingly, *Fgf23* and *Opn* mRNA expression remained unchanged between the genotypes in all three fractions, confirming an earlier report that osteoblastic Fgf23 production is not different between WT and *Hyp* mice [39]. The mRNA expression of *Fgfr1* and *Fgfr3* was lower in *Hyp*- versus WT-derived F-3 and/or F-5. In contrast, the mRNA abundance of *Ank*, *Enpp1*, *Opn*, *Fgf23*, *Fgfr1*, and *Fgfr3* was distinctly increased in *Hyp*-derived osteocyte-rich fractions F-6/7 and/or F-8/9 (Fig 2C). Most interestingly, *Tnap* mRNA abundance was decreased by 80%–90% in *Hyp*-derived osteocyte-rich fractions F-6/7 and F-8/9, relative to WT-derived osteocytes (Fig 2C). In accordance with the mRNA data, western blotting analysis of the pooled protein samples showed unchanged TNAP protein abundance in osteoblast-rich fractions F-3/4/5 but lower TNAP protein abundance in osteocyte-rich fractions F-6/7/8/9 isolated from *Hyp* mice, relative to WT controls (Fig 2D).

Collectively, these results corroborate the notion that osteocytes are the major source of the increased circulating Fgf23 levels in *Hyp* mice. Furthermore, our data show that there is not only increased OPN expression, but also increased mRNA expression of PPi-regulating factors such as *Ank* and *Enpp1* in *Hyp*-derived osteocytes. Collectively, the observed changes in gene expression of *Ank*, *Enpp1*, and *Tnap* in *Hyp*-derived osteocyte-rich cell fractions are able to explain the accumulation of PPi in the ECM of *Hyp* bones.

### Osteocyte-Like Cells Isolated from *Hyp* Mice Show Cell Autonomous Suppression of *TNAP* mRNA Expression and of Phosphate Production

**Fig 3:**
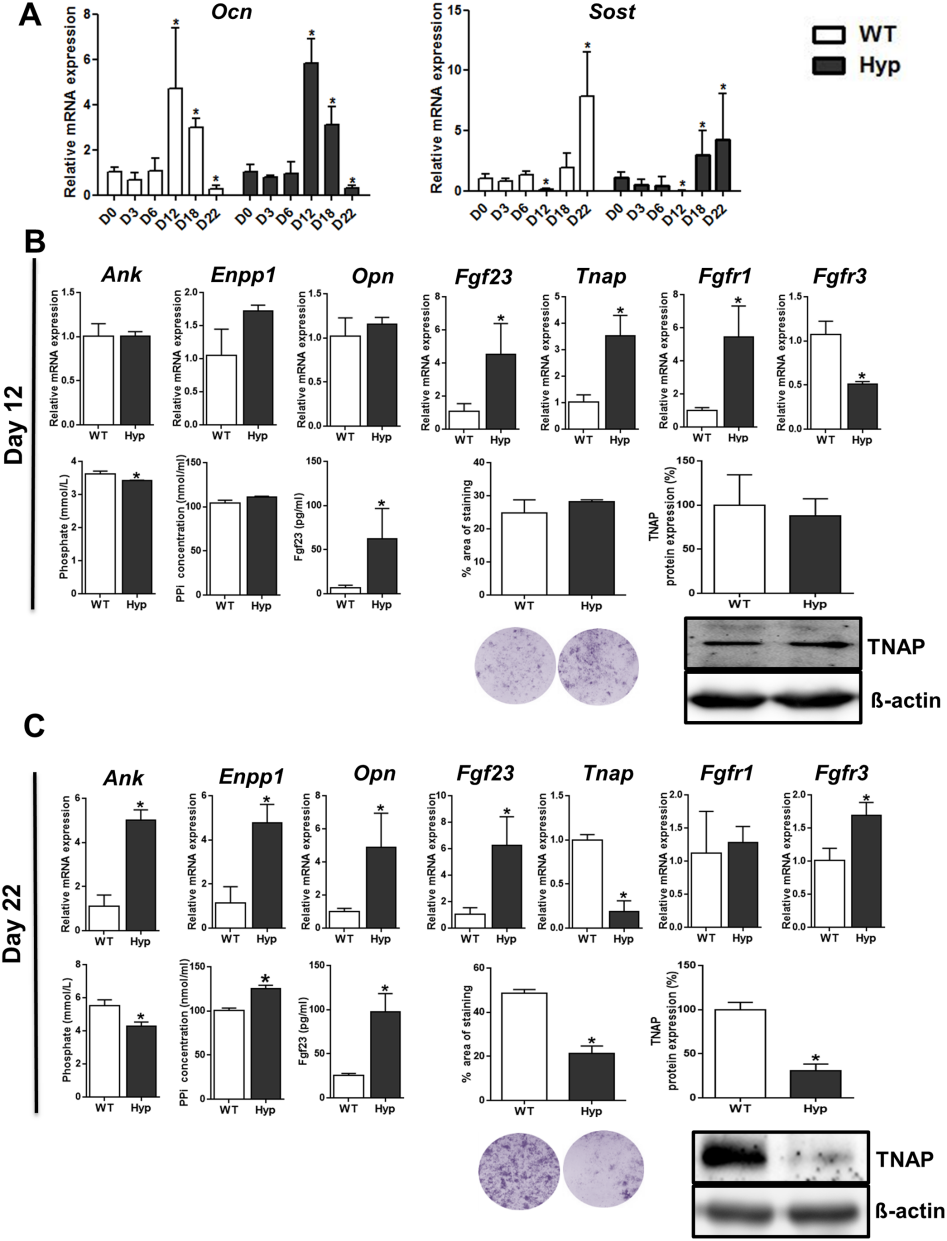
Osteocyte-like cells isolated from *Hyp* mice display decreased *Tnap* mRNA expression together with PPi accumulation in vitro. **(A)** mRNA abundance of the osteoblast-specific gene *Ocn* and of the osteocyte-specific gene *Sost* in calvarial cells isolated from newborn WT and *Hyp* mice and differentiated for 0–22 d (D0–D22). **(B–C)** mRNA abundance of *Ank*, *Enpp1*, *Opn*, *Fgf23*, *Tnap*, *Fgfr1*, and *Fgfr3* as well as concentration of Pi, PPi, and intact Fgf23 in cell culture supernatant, percentage NBT/BCIP-stained area, and TNAP protein expression in calvarial cells isolated from newborn WT and *Hyp* mice and differentiated for 12 d (differentiated osteoblasts) **(B)** or 22 d (osteocyte-like cells) **(C)**. Each data point is the mean ± SD of triplicates from four different animals. Individual values are given in S1 Data. *, *p* < 0.05 versus D0 in A; *, *p* < 0.05 versus WT in B and C.

To further examine whether osteoblasts and osteocytes isolated from *Hyp* mice differentially express PPi-regulating genes and *Tnap* in a cell autonomous fashion, we moved from the above mentioned ex vivo approach to an in vitro model. To this end, we isolated calvarial osteoblasts from newborn WT and *Hyp* mice, and differentiated the cells up to 22 d. At day 12, only little mineralized nodule formation was observed, and cells expressed maximum levels of *Ocn* mRNA, whereas at day 22, more mineralized nodules were formed and the mRNA expression of *Sost* was highest (Fig 3A). Therefore, we considered cells harvested at day 12 as differentiated osteoblasts and cells harvested at day 22 as osteocyte-like cells.

mRNA expression analysis at day 12 revealed no significant differences in *Ank*, *Enpp1*, and *Opn* expression between WT osteoblasts and *Hyp* osteoblasts (Fig 3B). *Fgfr1* mRNA abundance was up-regulated, whereas *Fgfr3* mRNA expression was lower in *Hyp* versus WT osteoblasts (Fig 3B). *Fgf23* mRNA abundance was higher in *Hyp* compared to WT osteoblasts already at day 12 (Fig 3B). We further analyzed if this increase in *Fgf23* mRNA expression led to increased Fgf23 secretion in the cell culture medium. We found ~60-fold higher concentrations of intact Fgf23 in the culture medium of *Hyp* osteoblasts (Fig 3B). Despite increased *Fgf23* mRNA expression and secretion, *Tnap* mRNA expression was increased in *Hyp* compared with WT osteoblasts (Fig 3B). Assessment of TNAP protein expression using western blotting analysis and of TNAP enzyme activity using BCIP/NBT staining showed similar levels of protein expression and activity in WT and *Hyp* osteoblasts at day 12 (Fig 3B). It is well known that TNAP is responsible for Pi production in vitro during differentiation by cleaving β–glycerophosphate, a component of the differentiation medium [42]. Therefore, we assessed Pi concentration in the culture medium as readout for TNAP enzyme activity. Pi concentration in the medium of *Hyp* osteoblasts was lower than that of WT osteoblasts (Fig 3B). We currently don’t have a good explanation for the discrepancy between *Tnap* mRNA expression and enzyme activity at the 12-day time point in *Hyp*-derived osteoblasts. As another readout for TNAP enzyme activity, we analyzed PPi concentration in the cell culture medium. However, no significant changes in PPi concentration were observed between cell culture medium from WT and *Hyp* osteoblasts at day 12 (Fig 3B).

In analogy to the osteocyte-rich fractions isolated from *Hyp* femurs, mRNA abundance of *Ank*, *Enpp1*, *Opn*, *Fgf23*, and *Fgfr3* was increased, whereas mRNA expression of *Tnap* was decreased, in *Hyp* compared to WT osteocyte-like cells differentiated for 22 d (Fig 3C). BCIP/NBT staining and western blotting analysis confirmed the decreased TNAP protein expression and activity in *Hyp* versus WT osteocyte-like cells (Fig 3C). In accordance with decreased Tnap expression, Pi concentration was lower and PPi concentration was higher in the cell culture medium from *Hyp* versus WT osteocyte-like cells (Fig 3C). Similar to our findings in osteoblast-like cells, intact Fgf23 in the culture medium was increased in *Hyp*-derived osteocyte-like cells (Fig 3C). Taken together, our data suggest that the up-regulation in *Ank*, *Enpp1*, and *Opn*, as well as the downregulation in *Tnap* mRNA and protein expression are cell autonomous effects in *Hyp* osteocyte-like cells.

### Suppression of TNAP Activity and Up-regulation of *Fgfr3* mRNA Expression in *Hyp* Osteocytes In Vivo

**Fig 4:**
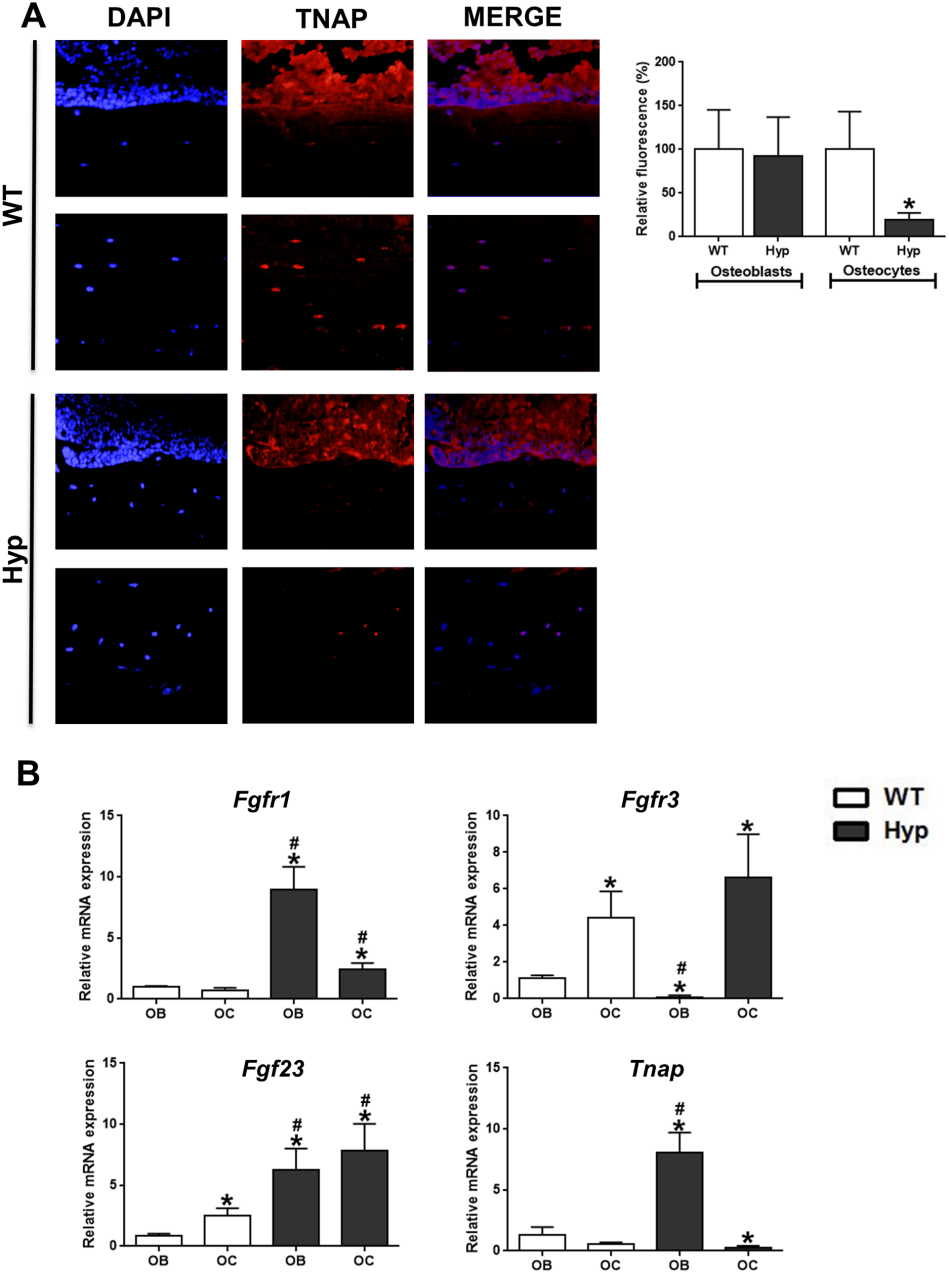
Decreased TNAP enzyme activity and up-regulated expression of *Fgfr3* mRNA in *Hyp* osteocytes in vivo. **(A)** Histochemical TNAP staining in undecalcified sections of distal femurs from WT and *Hyp* mice and quantification of relative fluorescence. Top panels for each genotype show endocortical bone surface, bottom panels cortical bone. Each data point is the mean ± SD of at least four mice. **(B)** In situ mRNA expression profiling of osteoblasts and osteocytes harvested by laser capture microdissection (LCM) in 4-μm-thick distal femoral cryosections. Each data point is the mean ± SD of three mice. Individual values are given in S1 Data. *, *p* < 0.05 versus WT osteocytes in A; *, *p* < 0.05 versus WT osteoblasts, #, *p* < 0.05 versus WT osteocytes in B.

To validate our ex vivo and in vitro finding that TNAP is suppressed in *Hyp*-derived osteocyte-like cells, we examined TNAP enzyme activity in osteoblasts and osteocytes in sections of femurs from WT and *Hyp* mice. As shown in Fig 4A, TNAP enzyme activity was profoundly suppressed in osteocytes, but not in osteoblasts, of *Hyp* compared with WT mice, corroborating the ex vivo and in vitro data.

A puzzling finding in our in vitro experiments with calvarial cells was that the up-regulated Fgf23 secretion observed in both osteoblast- and osteocyte-like cells from *Hyp* mice suppressed *Tnap* mRNA and protein abundance only in osteocyte-like cells but not in osteoblasts. To rule out that this finding was due to the calvarial origin of the cells, we isolated osteoblasts from femurs of newborn WT and *Hyp* mice and differentiated them for 12 and 22 d. Similar to calvarial cells, osteocalcin expression was higher at day 12, whereas *Sost* expression was higher at day 22 compared to day 12, consistent with a differentiated osteoblast-like phenotype at day 12 and an osteocyte-like phenotype at day 22 (S1A Fig). At day 12, femoral osteoblasts isolated from *Hyp* mice showed increased *Tnap* mRNA expression, decreased phosphate production, but unchanged BCIP/NBT staining relative to WT cells (S1B and S1C Fig). After 22 d of differentiation, *Tnap* mRNA abundance, phosphate production, and BCIP/NBT staining were decreased in *Hyp* versus WT cells (S1B and S1C Fig). *Fgf23* mRNA abundance was increased in *Hyp* versus WT cells at day 12 and 22 (S1B Fig). To test the differential sensitivity of calvarial versus femoral osteoblasts and osteocytes to recombinant FGF23 (rFGF23), we treated WT and osteoblasts and osteocyte-like cells with different doses of rFGF23, and monitored *Tnap* mRNA expression. The rFGF23-induced suppression of *Tnap* mRNA expression was generally similar in calvarial versus femoral osteoblasts and osteocyte-like cells from WT and *Hyp* mice (S2 Fig). However, in line with a lower sensitivity of *Hyp*-derived osteoblasts, higher concentrations of rFGF23 were needed to suppress *Tnap* mRNA in *Hyp* femoral and calvarial osteoblasts, relative to osteocytes (S2 Fig). Hence, the differences in TNAP expression and the response to pharmacological treatment with rFGF23 between osteoblasts and osteocyte-like cells isolated from *Hyp* mice were similar in cells derived from calvarial and femoral origin.

We previously reported that FGF23 inhibits *Tnap* transcription via FGFR3 [33]. The abovementioned increase in *Fgfr3* mRNA in *Hyp* osteocyte-rich fractions and osteocyte-like cells relative to WT cells would be consistent with the notion that the up-regulation of FGFR3 during osteocytic differentiation is the pivotal process making *Hyp* osteocytes more responsive to the suppressive effect of Fgf23 on *Tnap* transcription. To confirm the up-regulation of *Fgfr3* mRNA during osteocytic differentiation in vivo, we performed in situ mRNA expression analysis in frozen femur sections, employing laser capture microdissection (LCM), a technique which we recently developed [43]. We found that *Fgf23* mRNA abundance was ~3-fold higher in WT osteocytes than in WT osteoblasts (Fig 4B). Relative to WT osteoblasts, *Fgf23* mRNA expression was ~6–7-fold higher in *Hyp* osteoblasts and osteocytes (Fig 4B). In accordance with our in vitro and ex vivo data, *Tnap* mRNA expression was increased in *Hyp* osteoblasts, but suppressed in *Hyp* osteocytes, relative to WT osteoblasts and osteocytes, respectively (Fig 4B). *Fgfr1* mRNA expression was higher in osteoblasts and osteocytes of *Hyp* mice, relative to WT controls (Fig 4B). Notably, *Fgfr3* mRNA abundance was ~5-fold higher in WT osteocytes than in WT osteoblasts, whereas *Fgfr3* mRNA expression was suppressed in *Hyp* versus WT osteoblasts, but profoundly up-regulated in *Hyp* osteocytes (Fig 4B). *Hyp* osteocytes showed ~70-fold higher *Fgfr3* mRNA abundance than *Hyp* osteoblasts. Taken together, these findings support the notion that the distinct up-regulation in *Fgfr3* mRNA expression during osteocytic differentiation especially in *Hyp* mice is permissive to the Fgf23-mediated suppression of *Tnap* transcription in vitro and in vivo.

### Blocking of Fgf23—FGFR3 Signaling Increases *TNAP* mRNA Expression and Pi Production in *Hyp* Osteocyte-Like Cells In Vitro

**Fig 5:**
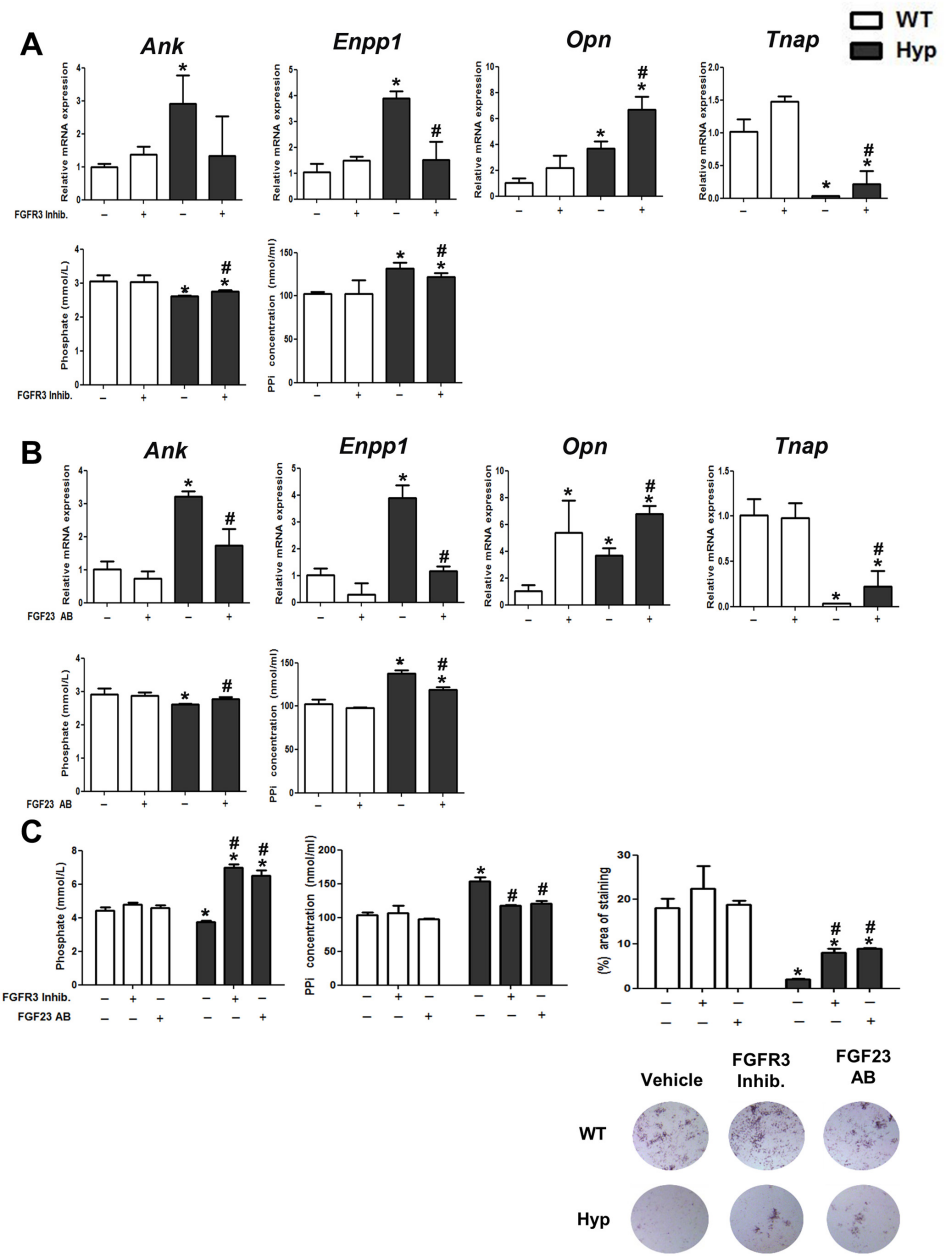
Inhibition of Fgf23-FGFR3 signaling increases *TNAP* expression and lowers PPi concentration in *Hyp*-derived osteocyte-like cells. **(A–B)** *Ank*, *Enpp1*, *Opn* and *Tnap* mRNA abundance as well as Pi and PPi concentration in cell culture supernatant of calvarial osteocyte-like cells differentiated for 22 d and treated for 24 h with a FGFR3 inhibitor **(A)** or anti-FGF23 antibody (FGF23 AB) **(B)**. **(C)** Phosphate and PPi concentration in cell culture supernatant as well as percent NBT/BCIP-stained area in cultures of calvarial osteocyte-like cells differentiated for 22 d, and treated daily over 4 d with a FGFR3 inhibitor or FGF23 AB. Each data point is the mean ± SD of four experimental samples. Individual values are given in S1 Data. *, *p* < 0.05 versus vehicle-treated WT cells, #, *p* < 0.05 versus vehicle-treated *Hyp* cells.

Next, we examined whether the changes in the mRNA expression of PPi-regulating genes in *Hyp* osteocyte-like cells are causatively linked to increased Fgf23 secretion. To this end, we isolated osteoblasts from newborn *Hyp* and WT mice, and treated osteocyte-like cells differentiated for 22 d with either neutralizing anti-FGF23 antibody (FGF23 AB) or an FGFR3 inhibitor for 24 h. In analogy to the experiments shown in Fig 3, *Hyp* osteocyte-like cells expressed distinctly lower *Tnap* mRNA, and showed lower Pi but higher PPi concentrations in cell culture medium, relative to WT cells (Fig 5A and 5B). With the exception of an up-regulation in *Opn* mRNA abundance, treatment of osteocyte-like cells with either FGFR3 inhibitor or FGF23 AB did not have significant effects in WT cells, but increased *Tnap* and *Opn*, and lowered *Ank* and *Enpp1* mRNA expression in *Hyp* osteocyte-like cells, relative to vehicle-treated cells (Fig 5A and 5B). However, both treatments did not restore *Tnap* mRNA expression in *Hyp* osteocyte-like cells to WT control levels. In concordance with the increased *Tnap* mRNA expression after inhibition of Fgf23 signaling, treatment of *Hyp* osteocyte-like cells with either FGFR3 inhibitor or FGF23 AB increased Pi and decreased PPi concentrations in the cell culture medium (Fig 5A and 5B).

Finally, to determine if longer term inhibition of Fgf23 signaling in *Hyp* osteocyte-like cells translates into a more complete correction of TNAP activity and PPi levels, we treated osteocyte-like cells with either FGFR3 inhibitor or FGF23 AB for 96 h and subsequently assessed PPi concentration and TNAP enzyme activity, using NBT/BCIP staining for the latter. As shown in Fig 5C, both treatments did not alter ALP staining or PPi concentration in WT osteocyte-like cells. However, treatment with either FGFR3 inhibitor or FGF23 AB increased ALP staining in *Hyp* osteocyte-like cells compared to vehicle-treated cells. The increase in TNAP activity was accompanied by a profound increase in Pi concentration and normalization of PPi levels in cell culture medium from *Hyp* osteocyte-like cells treated with either FGFR3 inhibitor or FGF23 AB (Fig 5C).

Taken together, our data provide evidence that increased Fgf23-FGFR3 signaling inhibits TNAP activity in *Hyp* osteocytes, causing PPi accumulation which in turn contributes to the mineralization defect observed in *Hyp* mice. However, inhibition of Fgf23 signaling did not completely normalize TNAP mRNA expression and enzyme activity, suggesting that other, still unknown factors are involved in the regulation of TNAP in *Hyp* osteocytes. Of note, inhibition of Fgf23 signaling increased phosphate concentrations in the cell culture medium beyond the levels found in WT osteocyte-like cells (Fig 5C). This finding may suggest that the increased phosphate production after inhibition of Fgf23 signaling could not be adequately used for mineralization in *Hyp* osteocyte-like cells due to the presence of additional inhibitors of mineralization, most likely OPN and ASARM peptides due to *Phex* deficiency.

### rFGF23 Suppresses *Tnap* mRNA Expression and Increases PPi in Wild-Type Osteocyte-Like Cells In Vitro and Bones of Wild-Type Mice In Vivo

**Fig 6:**
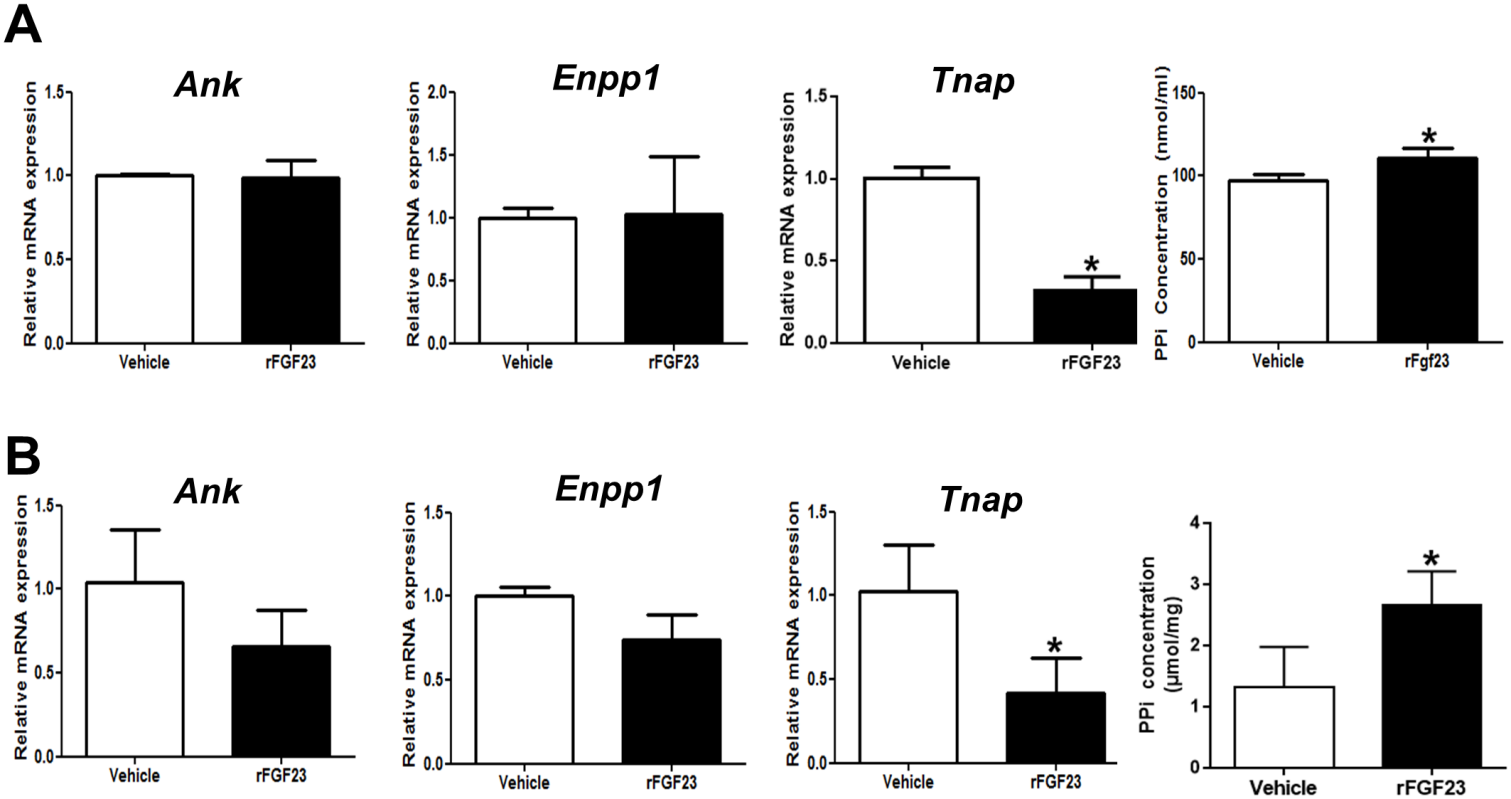
rFGF23 administration suppresses *Tnap* mRNA expression and increases PPi concentration in WT osteocyte-like cells and bones of WT mice. **(A)** mRNA abundance of *Ank*, *Enpp1*, and *Tnap* as well as PPi concentration in cell culture supernatant in WT calvarial osteocyte-like cells differentiated for 22 d, and subsequently treated with rFGF23 for 24 h. **(B)** mRNA abundance of *Ank*, *Enpp1*, and *Tnap* in total RNA isolated from whole femurs and PPi concentration in extracts of whole tibiae from 3-mo-old male WT mice treated with rFGF23 or vehicle for 5 d. Each data point is the mean ± SD of at least four experimental samples in A, and of at least five mice in B. Individual values are given in S1 Data. *, *p* < 0.05 versus vehicle.

*Hyp* osteocyte-like cells are not only characterized by suppressed *Tnap* expression but also by increased abundance of genes associated with PPi production such as *Enpp1* and *Ank*. To exclude the role of altered PPi production after inhibition of Fgf23 signaling in *Hyp* osteocyte-like cells, we treated WT osteocyte-like cells with rFGF23. We previously showed that treatment of WT osteoblasts with rFGF23 does not alter the expression of *Enpp1* and *Ank* [33]. Treatment of WT osteoblasts with rFGF23 suppressed *Tnap* mRNA expression and increased PPi concentrations in the culture medium, independent of changes in expression of *Enpp1* or *Ank* (Fig 6A).

To further validate the link between Fgf23, TNAP, and PPi in vivo, we treated WT mice with rFGF23. A 5-d treatment with rFGF23 suppressed *Tnap* mRNA expression (Fig 6B) and significantly increased PPi concentrations in bones of WT mice (Fig 6B). In line with our in vitro data, rFGF23 treatment did not alter mRNA levels of *Ank* and *Enpp1* in bones. Taken together, these data corroborate the notion that extracellular FGF23 is independently associated with PPi levels in bone through its suppressive effect on TNAP.

### Osteoblast Lineage Specific Deletion of *Fgf23* Rescues the Suppression of TNAP Activity in Osteocytes of *Hyp* Mice

**Fig 7:**
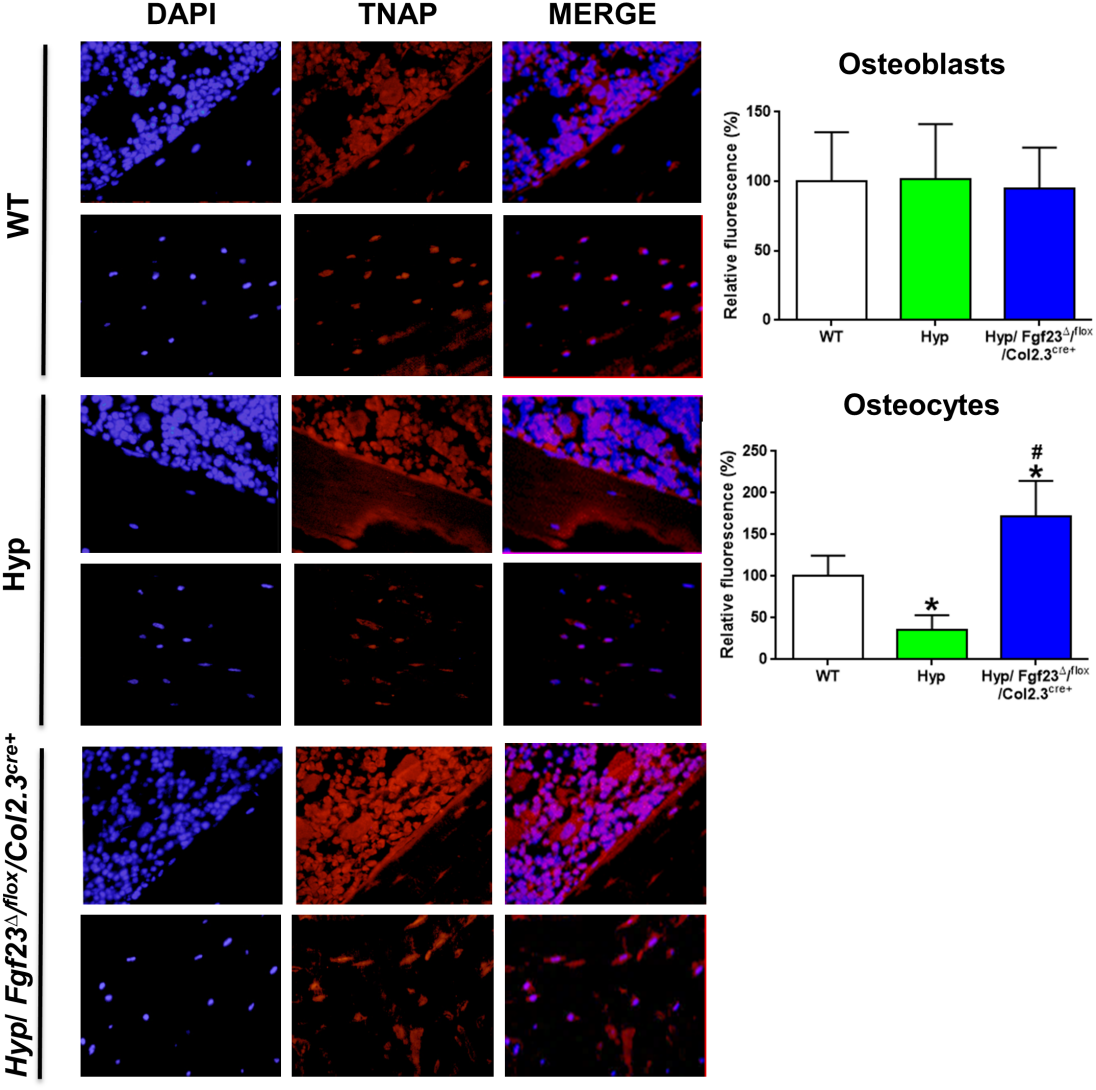
Bone specific deletion of *Fgf23* rescues suppressed TNAP activity in *Hyp* mice. Histochemical TNAP staining in bone sections from WT, *Hyp*, and *Hyp*/*Fgf23* ^*Δ/flox*^ */Col2*.*3* ^*cre+*^ mice and quantification of relative fluorescence in osteoblasts and osteocytes. Top panels for each genotype represent endocortical bone surface, bottom panels cortical bone. Each data point is the mean ± SD of at least four mice. Individual values are given in S1 Data. *, *p* < 0.05 versus WT; #, *p* < 0.05 versus *Hyp*.

Finally, to test whether excessive secretion of Fgf23 is responsible for the decreased Tnap expression and PPi accumulation in *Hyp* bones in vivo, we analyzed bones from *Hyp* mice in which *Fgf23* was specifically deleted in cells of the osteoblastic lineage. To this end, we used a novel mouse model (*Fgf23* ^Δ/flox^ */Col2*.*3* ^*cre+*^) carrying a germline-deleted *Fgf23* allele together with a floxed *Fgf23* allele. *Fgf23* ^Δ/flox^ mice were mated with type 1 collagen 2.3 kb promoter-cre mice, resulting in deletion of *Fgf23* in the osteoblast lineage [44]. *Fgf23* ^Δ/flox^ */Col2*.*3* ^*cre+*^ mice were mated with *Hyp* mice to obtain *Hyp*/*Fgf23* ^Δ/flox^ */Col2*.*3* ^*cre+*^ mice, a *Hyp* mouse model with conditional deletion of *Fgf23* in osteoblasts and osteocytes. Analysis of TNAP enzyme activity in femur sections of 3-mo-old WT, *Hyp*, and *Hyp*/*Fgf23* ^Δ/flox^ */Col2*.*3* ^*cre+*^ mice showed that the suppression of TNAP enzyme activity in *Hyp* osteocytes was rescued in *Hyp*/*Fgf23* ^Δ/flox^ */Col2*.*3* ^*cre+*^ mice (Fig 7). TNAP activity was similar in osteoblasts at the bone surface in all genotypes, corroborating the notion that Fgf23 does not contribute to the regulation of TNAP activity in osteoblasts of *Hyp* mice (Fig 7). In a subset of these mice, we were able to quantify PPi in distal femurs. Bone PPi concentration was 1.18 ± 0.009 μmol/mg in WT (*n* = 4), 1.49 ± 0.007 μmol/mg in *Hyp* (*n* = 2), and 0.91 ± 0.002 μmol/mg in *Hyp*/*Fgf23* ^Δ/flox^ */Col2*.*3* ^*cre+*^ mice (*n* = 2). Collectively, these data suggest that increased Fgf23 secretion is indeed responsible for the suppression of TNAP expression and subsequent PPi accumulation in *Hyp* bones.

## Discussion

**Fig 8:**
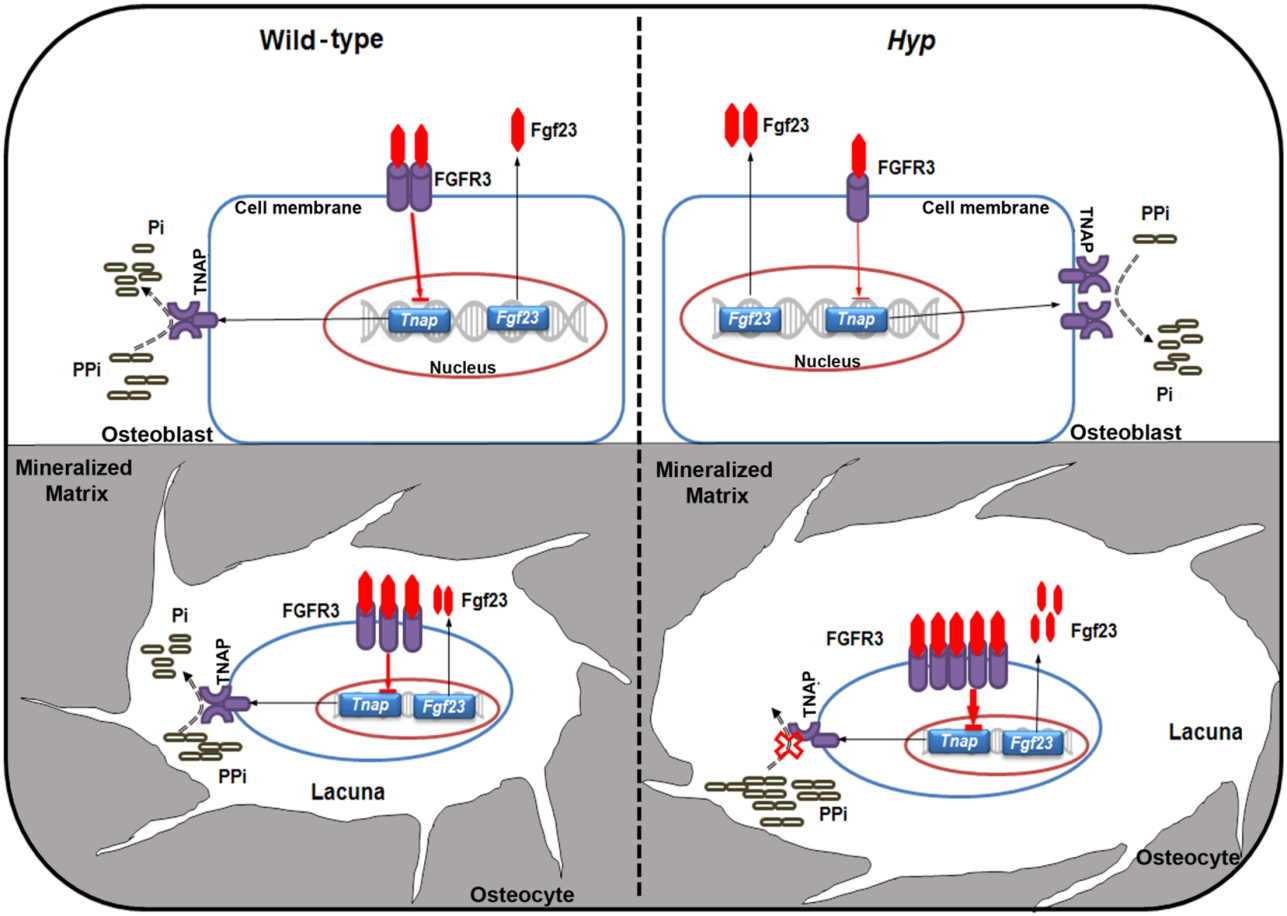
Proposed model of PPi accumulation through increased Fgf23-FGFR3 signaling in *Hyp* osteocytes. Fgf23 secreted into the extracellular fluid binds to FGFR3 and acts as a transcriptional suppressor of *Tnap*. TNAP is a central molecule in the mineralization process and favors mineralization through hydrolyzing PPi, thus providing Pi for mineralization. Fgf23 secretion is up-regulated in osteoblasts and especially osteocytes in *Hyp* bones, relative to WT osteoblasts and osteocytes. FGFR3 expression is reduced in *Hyp* compared with WT osteoblasts. However, differentiation of osteoblasts into osteocytes is associated with a distinct up-regulation of FGFR3 expression especially in *Hyp* bones. Together with the increased secretion of Fgf23, the upregulation in FGFR3 leads to autocrine/paracrine suppression of TNAP activity in *Hyp* osteocytes but not osteoblasts, causing accumulation of PPi and inhibition of bone mineralization in osteocyte lacunae.

In the current study, we identified a novel mechanism contributing to the defective mineralization in *Hyp* mice. Our data indicate that besides its endocrine role as phosphaturic hormone, excessive osteocytic Fgf23 secretion has an additional para-/autocrine role in the development of osteomalacia in *Hyp* mice by suppressing TNAP activity in osteocytes, which in turn leads to accumulation of PPi, a potent inhibitor of mineralization. We hypothesize that the cell-specific suppression of Tnap in osteocytes but not osteoblasts of *Hyp* mice is based upon the profound up-regulation of *Fgfr3* expression during osteocytic differentiation. This model is shown in Fig 8. Moreover, we demonstrated that conditional deletion of *Fgf23* in cells of the osteoblastic lineage rescued the suppressed TNAP activity in osteocytes of *Hyp* mice in vivo, and that blocking of the cell-autonomous increase in Fgf23-FGFR3 signaling in *Hyp*-derived osteocyte-like cells improved TNAP activity and phosphate production, and decreased PPi concentration in vitro. However, inhibition of Fgf23 signaling did not fully correct the mineralization defect in vitro, suggesting that increased local Fgf23 production is only partially responsible for impaired mineralization in *Hyp* mice.

The XLH and *Hyp* phenotypes are caused by loss-of-function mutations in *PHEX/Phex*. It was previously thought that increased ASARM peptides were largely responsible for the cell autonomous mineralization defect observed in osteoblasts isolated from *Hyp* mice, and partially for the osteomalacia found in *Hyp* mice [18]. Phex binds to and proteolytically cleaves free ASARM peptides [45,46], and also degrades OPN [21]. ASARM peptides and OPN are increased in bones of *Hyp* mice [19], and known to impair mineralization in vivo and in vitro [45,47]. It is interesting to note in this context that transgenic overexpression of *PHEX* under different promoters only partially rescued the osteomalacia in *Hyp* mice (PHEX-tg/*Hyp*) [8,48]. The current study may provide a possible explanation why osteomalacia was not fully corrected in the majority of studies with PHEX-tg/*Hyp* mice. In this regard, Fgf23 levels remained significantly higher in Phex-Tg/*Hyp* mice [49], thus TNAP activity may have remained suppressed in these mice, causing impaired mineralization via PPi accumulation.

An interesting aspect of our study was the striking differences between the expression profiles of osteoblasts and osteocytes isolated by sequential digestion from *Hyp* bones. Whereas *Tnap* mRNA and protein expression was strongly suppressed in *Hyp*-derived osteocyte-like cells, *Tnap* mRNA expression was ~50-fold increased in *Hyp* relative to WT osteoblast-like cells. Vice versa, we found increased Fgf23 expression only in osteocytes but not in osteoblasts of *Hyp* mice as evidenced by sequential digestion of bones. This finding is in agreement with earlier reports [29,39]. However, LCM-based in situ expression profiling of osteoblasts and osteocytes revealed increased *Fgf23* mRNA abundance not only in osteocytes, but also in osteoblasts in *Hyp* bones. Therefore, the relative contribution of osteoblasts and osteocytes to the increased circulating Fgf23 levels in *Hyp* mice is not entirely clear. Furthermore, in agreement with earlier studies [39], we found that *Fgfr3* mRNA abundance is profoundly up-regulated during osteocytic differentiation especially in *Hyp* mice, supporting the notion that the higher membrane abundance of FGFR3 in *Hyp* osteocytes versus osteoblasts forms the basis for the cell type-specific suppression of *Tnap* transcription by Fgf23. Collectively, these results underscore the biological differences between osteocytes and osteoblasts in *Hyp* mice and suggest that the increased ALP activity in the serum of *Hyp* mice and XLH patients more likely reflects changes in bone surface cells rather than osteocytes. Although we did not assess TNAP expression in newly embedded osteocytes at bone-forming surfaces, we speculate that suppression of TNAP with subsequent accumulation of PPi may also occur in osteoid seams at the bone surface, not only in osteocyte lacunae. Up-regulation of FGFR3 expression in newly embedded osteocytes may lead to Fgf23-mediated suppression of TNAP, which may in turn result in accumulation of PPi and subsequent inhibition of mineralization in the widened osteoid seams of *Hyp* mice in addition to increased concentrations of ASARM peptides and OPN.

TNAP, an ectoenzyme, is responsible for the local production of Pi for mineralization via hydrolyzing PPi in the ECM. *Tnap* loss-of-function mutants are characterized by impaired bone mineralization via accumulation of PPi [50]. Furthermore, TNAP-deficient osteoblasts fail to mineralize in vitro [51], underscoring the pivotal importance of TNAP for bone mineralization. We previously reported that Fgf23-FGFR3 signaling suppresses TNAP transcription and activity, causing PPi accumulation and inhibition of mineralization in vitro [33]. Here, we showed that inhibition of Fgf23-FGFR3 signaling in *Hyp* osteocyte-like cells by treatment with either an FGFR3 inhibitor or an anti-FGF23 antibody improved TNAP activity and decreased PPi concentration in vitro. In addition, bone-specific deletion of *Fgf23* in *Hyp*/*Fgf23* ^Δ/flox^ */Col2*.*3* ^*cre+*^ mice rescued the suppressed TNAP activity in osteocytes of *Hyp* mice. Although bony PPi concentrations were not quantified after treatment of *Hyp* mice with anti-Fgf23 antibodies [9] or a pan-FGFR inhibitor [24], it is likely that systemic anti-FGF23 treatment or pan-FGFR inhibition also, at least partially, corrects the increased PPi concentration in bone. This idea is indirectly supported by our finding that a 5-d treatment of WT mice with rFGF23 suppressed TNAP expression and increased PPi in bone, suggesting that circulating FGF23 levels are able to modulate bony PPi metabolism. The latter findings may also have implications for tumor-induced osteomalacia (TIO), because our data suggest that excessive extraskeletal production of FGF23 may also lead to PPi accumulation in bone. However, due to the low affinity of the FGFR3 signaling pathway [33], this mechanism may only become operative at high circulating FGF23 levels.

Vitamin D hormone levels are inappropriately low in *Hyp* mice and in XLH patients due to the FGF23-mediated suppression of renal 1α-hydroxylase [15]. The vitamin D hormone not only governs intestinal absorption of calcium and phosphate [52], but also inhibits bone mineralization by stimulating the transcription of *Enpp1*, *Ank*, and *Opn* [33,35]. In line with low vitamin D hormone levels in *Hyp* mice, the mRNA abundance of *Ank* and *Enpp1* was almost undetectable in *Hyp*-derived F-3 osteoblasts. On the contrary, the mRNA abundance of *Ank* and *Enpp1* was higher in *Hyp*- than WT-derived osteocyte-rich cell fractions ex vivo. Therefore, inappropriately low vitamin D hormone levels cannot account for the changes observed in *Ank* and *Enpp1* expression in *Hyp*-derived osteocyte-like cells. Furthermore, inhibition of Fgf23 signaling partially corrected the increased *Ank* and *Enpp1*, but not the increased *Opn* mRNA expression, in *Hyp*-derived osteocyte-like cells in our experiments. Collectively, our data and the work of others suggest that *Phex* deficiency [39,53], via only partially known signaling pathways at present time, induces a complex pattern of altered gene regulation in which increased Fgf23 transcription is only a portion of the pathophysiology.

In conclusion, we have found that the mineralization defect in bones of *Hyp* mice and in cultures of *Hyp*-derived osteoblasts is not only due to local accumulation of ASARM peptides and OPN but also due to the Fgf23-driven accumulation of PPi, another potent mineralization inhibitor. Clearly, more work is required to disentangle the complex interactions between *Phex* deficiency, Fgf23 secretion, and para-/autocrine Fgf23 feedback signaling in osteocytes of *Hyp* mice. A more complete understanding of these aspects of osteocyte biology may help to design novel treatments for the mineralization defects observed in diseases associated with excessive osteocytic Fgf23 secretion such as XLH or chronic kidney disease.

## Materials and Methods

### Animals

All animal studies were approved by the Ethical Committee of the University of Veterinary Medicine, Vienna and by the Austrian Federal Ministry of Science and Research and were undertaken in strict accordance with prevailing guidelines for animal care and welfare (permit number BMWF-68.205/0037-II/3b/2013). Both WT controls and *Hyp* mice were on C57BL/6 background and were kept on normal mouse chow (Ssniff, Soest, Germany). As described [44], a conditional *Fgf23* mouse model that harbored alleles with floxed exon 2 was developed through standard gene targeting. An *Fgf23* null allele (Δ) created by mating to the global *ella*-cre transgenic line was bred onto the flox-*Fgf23* background to produce *Fgf23* ^Δ/flox^ mice. *Fgf23* ^Δ/flox^ mice were crossed with type 1 collagen 2.3-kb promoter-cre mice, resulting in *Fgf23* ^Δ/flox^ */Col2*.*3* ^*cre+*^ mice by standard mating strategies; this line was mated onto the *Hyp* genetic background to obtain *Hyp*/*Fgf23* ^Δ/flox^ */Col2*.*3* ^*cre+*^ mice [44]. Genotyping of the mice was performed by multiplex PCR using genomic DNA extracted from the tail. The mice were kept at 24°C with a 12 h/12 h light/dark cycle and were allowed free access to food and tap water. All experiments were performed on 3-mo-old males. Some WT mice received daily intraperitoneal injections of vehicle (phosphate-buffered saline with 2% DMSO) or 10 μg recombinant human FGF23 R176/179Q (rFGF23, kindly provided by Amgen, Thousand Oaks, CA) per mouse for 5 days, and were killed 8–12 hours after the last injection. At necropsy, the mice were exsanguinated from the abdominal vena cava under anesthesia with ketamine/xylazine (67/7 mg/kg i.p.) for collection of serum and bones.

### Biochemical Analyses

Serum calcium, phosphorus, and ALP activity were analyzed using a Cobas c111 analyzer (Roche). Intact Fgf23 in serum and culture medium was determined by ELISA (Immutopics).

### Isolation of Osteoblast-Rich and Osteocyte-Rich Fractions from Femurs

Primary osteoblast-rich and osteocyte-rich cell fractions were isolated as previously described [54]. Briefly, both femurs were collected, carefully defleshed, the epiphysis was cut off, and bone marrow was flushed out using HBSS calcium-free and magnesium-free medium (Life Technologies). Subsequently, the washed femurs were minced into small pieces using scissors and digested with 1.25 mg/ml type II collagenase (Invitrogen) at 37°C. Cells released after the first two digestions of 15 min each were discarded. Cells released after the next three consecutive digestions of 20 min each were collected after passing through a 100-μm nylon cell strainer as Fraction 3 (F-3), Fraction 4 (F-4) and Fraction 5 (F-5), respectively. Digested bones were washed once, and treated with 4 mM EGTA in HBSS calcium-free and magnesium-free medium for 20 min at 37°C. Cells released after this treatment were collected, and bones were again digested using 1.25 mg/ml type II collagenase for 20 min at 37°C. Cells released after this digestion were collected and combined with the previous fraction and named F-6/7. Thereafter, bones were again treated with 4 mM EGTA for 20 min and subsequently with 1.25 mg/ml type II collagenase for 20 min, cells were collected as before, and named F-8/9.

### LCM

LCM was performed as described previously [43]. Briefly, distal femurs of 3-mo-old WT and *Hyp* mice were snap-frozen in liquid nitrogen with OCT compound (Sakura Finetek, Zoeterwoude, Netherlands). Four-μm-thick cryosections of were cut on a cryotome (Leica Kryostat 1720), using the cryotape method as described [55]. Cryosections were quickly stained with HistoStain (Arcturus). Osteoblasts and osteocytes (~100–200 cells per sample each) in the cancellous bone of the distal femoral metaphysis were dissected based on their typical morphology, using a Veritas (Arcturus) LCM system.

### RNA Isolation and Quantitative Real-Time PCR

Total RNA was isolated directly after collection of the bone cell fractions using Tri-Reagent (Ambion, Thermo Fisher Scientific) according to the manufacturer's protocol. RNA quantity was determined using a Nanodrop photometer (Thermo Scientific). For LCM-harvested osteoblasts and osteocytes, total RNA was extracted using the SPLIT RNA Extraction Kit (Lexogen), and RNA quality was determined by Agilent RNA 6000 Pico Chip (Agilent Technologies). cDNA synthesis was performed using the High capacity cDNA reverse transcription kit (Applied Biosystems). Quantitative RT-PCR was performed on a Rotor-Gene 6000 (Corbett Life Science) using 5X HOT Firepol Evagreen qPCR mix plus (Solis BioDyne). A melting curve analysis was done for all assays. Primer sequences are available on request. Efficiencies were examined based on a standard curve. Expression of target genes was normalized to the expression of the housekeeping gene glyceraldehyde-3-phosphate-dehydrogenase (*Gapdh*).

### Bone Histology

Isolated mouse femurs were fixed in 4% paraformaldehyde at 4°C overnight and were processed and embedded in methylmethacrylate as described previously [56]. Midsagittal sections of the distal femurs were prepared using a HM 355S microtome (Microm, Walldorf, Germany), and were stained with von Kossa/McNeal [57]. Sections were analyzed using a Zeiss Axioskop II microscope.

### Culture of Osteoblast- and Osteocyte-Like Cells and In Vitro Experiments

Calvariae were aseptically harvested from 3-d-old mice, minced and incubated with digestion medium (α-MEM medium, 2 mg/ml type II collagenase (Invitrogen) and 2% Penicillin-Streptomycin) at 37°C in a water bath for 4 h. Bone fragments were washed with PBS and cultured in α-MEM medium supplemented with 2% Penicillin-Streptomycin and 10% calf serum (PAA). A similar protocol was followed using femora of 3-d-old mice to obtain femural osteoblast cultures. After 90% confluence, cells were grown in the presence of osteoblastic differentiation medium (50 μg/ml ascorbic acid and 10 mM β-glycerophosphate) for 12–22 d as specified. The differentiated cells were treated with various concentrations of recombinant human FGF23 carrying the R176/179Q stabilizing mutations (rFGF23, kindly provided by Amgen Inc., Thousand Oaks, CA, US) for 24 h, 20 ng/ml rat anti-FGF23 antibody (kindly provided by Amgen Inc., Thousand Oaks, CA, US), or 25 nM FGFR3 inhibitor PD173074 (Sigma) for 24 or 96 h. At the various time points following treatment, cell culture supernatant and samples for RNA isolation were collected and stored at −80°C. For BCIP/NBT staining, cells were fixed using acetone and methanol (30:70) for 5 min at −20°C, and stained using TNM buffer (100 mM Tris-HCl, pH 9.5, 100 mM NaCl, 5 mM MgCl2) containing 0.175 mg/mL 3-bromo-4-chloro-3-indolyl phosphate (BCIP, Sigma) and 0.45 ng/mL nitrotetrazolium blue chloride (NBT, Sigma) for 45 min at room temperature. Stained cells were photographed using a stereomicroscope (Stemi SV6; Zeiss), and the percent area of positive staining was measured using Image J software.

### Histochemistry and Immunohistochemistry

For immunohistochemistry, 5-μm-thick undecalcified sections were obtained from plastic-embedded femurs as described [56]. Sections were deplastified, incubated for 15 min in 3% hydrogen peroxide in PBS to block endogenous peroxidase activity, and, after blocking with 10% rabbit serum, incubated with rabbit anti-OPN antibody (Abcam, 1:300) at 4°C overnight. After washing, sections were incubated for 2 h with biotinylated goat anti-rabbit secondary antibody (1:2,000, Vector). Finally, the sections were counterstained with Mayer's hematoxylin. Negative control was performed by omitting the primary antibody. For TNAP staining, deplastified bone sections were incubated with vector red ALP staining kit (Vector Laboratories) according to the manufacturer's protocol. Fluorescent images of TNAP and DAPI were obtained using appropriate filter sets. Fluorescence measurements were obtained using Image J software as described previously [58]. Fluorescence along the bone surface was marked manually and quantified using Image J for obtaining relative fluorescence of osteoblasts. At least 15 osteocytes per image and a total of 6 images per animal were chosen for the quantification of relative fluorescence in osteocytes. Relative fluorescence in osteocytes was normalized to cell number. The sections were analyzed using a Zeiss Axioskop 2 microscope.

### Protein Isolation from Bone

Proteins from femurs were isolated using a previously described protocol [59]. Briefly, femurs were carefully defleshed and bone marrow was flushed out. After demineralization (300 μl of 1.2 M HCl at 4°C overnight), proteins were isolated using 6M guanidine-HCL in 100 mM Tris buffer, pH 7.4, at 4°C for 72 h. Extracted proteins were concentrated using ethanol precipitation and re-dissolved in 8M urea buffer. Protein concentration was determined using a BCA assay (Thermo Scientific).

### Western Blotting

Proteins were solubilized in Laemmli sample buffer, fractionated on SDS–PAGE (50 μg/well), and transferred to a nitrocellulose membrane (Thermo Scientific). Immunoblots were incubated overnight at 4°C with polyclonal rabbit anti‐OPN (1:2,000, Abcam), polyclonal goat anti-TNAP (1:2,000, R&D Systems) and monoclonal mouse anti-β-actin (1:5,000, Sigma) in 2% (w/v) bovine serum albumin (BSA, Sigma) in a TBS‐T buffer [150 mM NaCl, 10 mM Tris (pH 7.4/HCl), 0.2% (v/v) Tween-20]. After washing, membranes were incubated with horseradish peroxidase-conjugated secondary antibodies (Sigma). Specific signal was visualized by ECL kit (Amersham Life Sciences). The protein bands were quantified by Image Quant 5.0 software (Molecular Dynamics).

### Quantification of PPi Levels

PPi was extracted from whole femurs with 1.2 M HCl at 4°C overnight. HCl was evaporated at 99°C, and samples were resuspended in deionized water. The amount of PPi extracted from bone or in cell culture supernatant was quantified using the PPiLight Inorganic Pyrophosphate Assay (LONZA) according to the manufacturer's protocol. Sodium PPi tetrabasic decahydrate (Sigma) was used as standard.

### Statistical Analysis

Statistics were computed using PASW Statistics 17.0 (SPSS Inc., Chicago, IL, US). The data were analyzed by two-sided *t* test (two groups) or one-way analysis of variance (ANOVA) followed by Student-Newman-Keuls multiple comparison test (>2 groups). *p*-Values of less than 0.05 were considered significant. Data represent mean values ± SD.

## Supporting Information

S1 Data Data used for making graphs for “Excessive osteocytic Fgf23 secretion contributes to pyrophosphate accumulation and mineralization defect in *Hyp* mice.” (XLSX)

S1 Fig Osteocyte-like cells isolated from femurs of newborn *Hyp* mice show decreased *Tnap* mRNA expression and diminished TNAP activity in vitro. **(A)** mRNA abundance of the osteoblast-specific gene *Ocn* and of the osteocyte-specific gene *Sost* in femoral cells isolated from newborn WT and *Hyp* mice and differentiated for 12 (differentiated osteoblasts, D12) or 22 d (osteocyte-like cells, D22). **(B)** mRNA abundance of *Tnap*, *Fgf23*, and concentration of inorganic phosphate in cell culture supernatant, and **(C)** BCIP/NBT staining in femoral cells isolated from newborn WT and *Hyp* mice and differentiated for 12 d or 22 d. Each data point is the mean ± SD of four experimental samples. Individual values are given in S1 Data. *, *p* < 0.05 versus D12 in A, *, *p* < 0.05 versus WT.
(TIF)

S2 Fig Suppression of *Tnap* mRNA expression by rFGF23 in calvarial and femoral osteoblasts and osteocyte-like cells isolated from WT and *Hyp* mice. **(A–B)** Effects of rFGF23 treatment for 24 h in calvarial **(A)** and femoral **(B)** osteoblast-like cells (differentiated for 12 d, D12) and osteocyte-like cells (differentiated for 22 d, D22) isolated from newborn WT and *Hyp* mice. Each data point is the mean ± SD of triplicates from three different animals. Individual values are given in S1 Data. *, *p* < 0.05 versus vehicle.
(TIF)

## Abbreviations

ALP: alkaline phosphatase
ANK: progessive ankylosis
ASARM: acidic serine- and aspartate-rich MEPE-associated motif
*Dmp-1*: Dentin matrix protein-1
ECM: extracellular matrix
ENPP1: ectonucleotide pyrophosphatase/phosphodiesterase 1
FGF23: fibroblast growth factor-23
HA: hydroxyapatite
LCM: laser capture microdissection
MEPE: matrix extracellular phosphoglycoprotein
*Ocn*: osteocalcin
OPN: osteopontin
*PHEX*: phosphate-regulating gene with homologies to endopeptidases on the X-chromosome
Pi: inorganic phosphate
PPi: pyrophosphate
rFGF23: recombinant FGF23
*Sost*: sclerostin
TIO: tumor-induced osteomalacia
TNAP: tissue nonspecific alkaline phosphatase
WT: wild-type
XLH: X-linked hypophosphatemia
