# Supplementary figures and images for: Excessive Osteocytic Fgf23 Secretion Contributes to Pyrophosphate Accumulation and Mineralization Defect in Hyp Mice
Source: PLoS Biol. 2016 Apr 1;14(4):e1002427. doi: 10.1371/journal.pbio.1002427 (PMC4818020; doi:10.1371/journal.pbio.1002427)

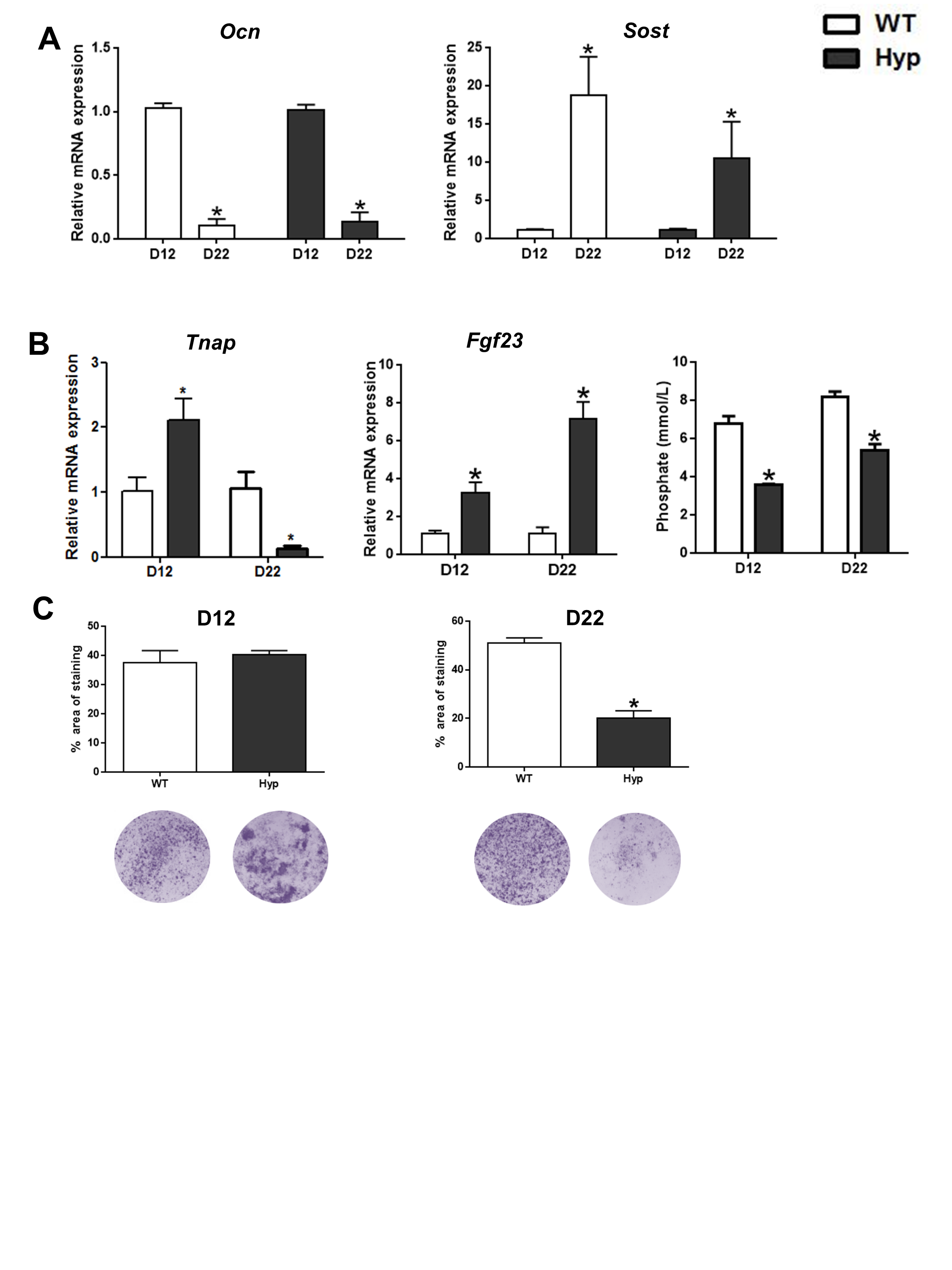

Supplement: S1 Fig — (A) mRNA abundance of the osteoblast-specific gene Ocn and of the osteocyte-specific gene Sost in femoral cells isolated from newborn WT and Hyp mice and differentiated for 12 (differentiated osteoblasts, D12) or 22 d (osteocyte-like cells, D22). (B) mRNA abundance of Tnap, Fgf23, and concentration of inorganic phosphate in cell culture supernatant, and (C) BCIP/NBT staining in femoral cells isolated from newborn WT and Hyp mice and differentiated for 12 d or 22 d. Each data point is the mean ± SD of four experimental samples. Individual values are given in S1 Data. *, p < 0.05 versus D12 in A, *, p < 0.05 versus WT. (TIF) [file pbio.1002427.s002.tif]

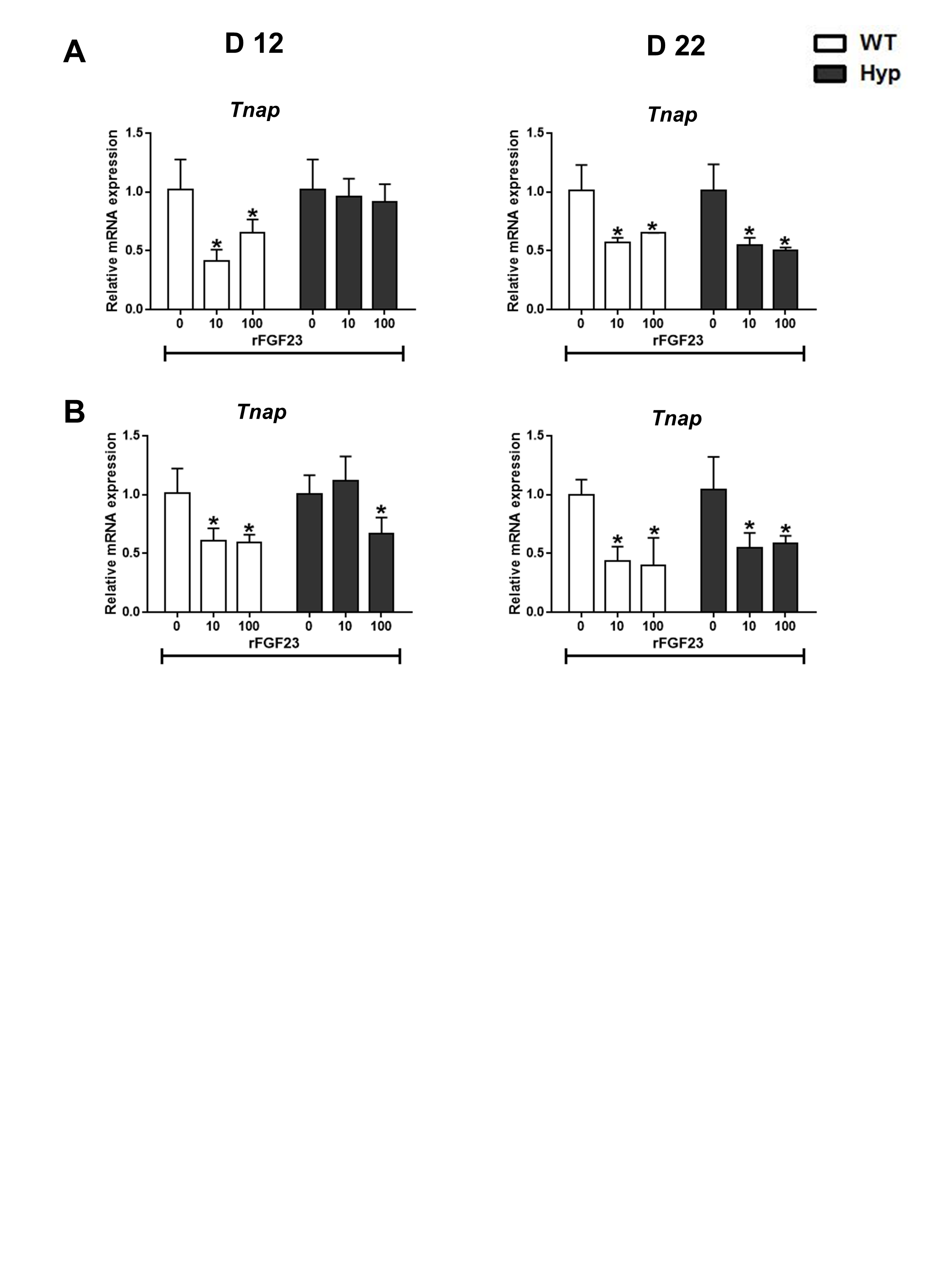

Supplement: S2 Fig — (A–B) Effects of rFGF23 treatment for 24 h in calvarial (A) and femoral (B) osteoblast-like cells (differentiated for 12 d, D12) and osteocyte-like cells (differentiated for 22 d, D22) isolated from newborn WT and Hyp mice. Each data point is the mean ± SD of triplicates from three different animals. Individual values are given in S1 Data. *, p < 0.05 versus vehicle. (TIF) [file pbio.1002427.s003.tif]
